# *Paenibacillus* as a Biocontrol Agent for Fungal Phytopathogens: Is *P. polymyxa* the Only One Worth Attention?

**DOI:** 10.1007/s00248-024-02450-8

**Published:** 2024-10-31

**Authors:** Jakub Dobrzyński, Aleksandra Naziębło

**Affiliations:** Institute of Technology and Life Sciences – National Research Institute, Al. Hrabska 3, 05-090 Raszyn, Poland

**Keywords:** Fungal phytopathogens, *Paenibacillus*, PGPB, Biocontrol

## Abstract

Control of fungal phytopathogens is a significant challenge in modern agriculture. The widespread use of chemical fungicides to control these pathogens often leads to environmental and food contamination. An eco-friendly alternative that can help reduce reliance on these chemicals is plant growth–promoting bacteria (PGPB), particularly those of the genus *Paenibacillus*, which appear to be highly effective. The review aims to summarize the existing knowledge on the potential of *Paenibacillus* spp. as fungal biocontrol agents, identify knowledge gaps, and answer whether other species of the genus *Paenibacillus*, in addition to *Paenibacillus polymyxa*, can also be effective biocontrol agents. *Paenibacillus* spp. can combat plant phytopathogens through various mechanisms, including the production of lipopeptides (such as fusaricidin, paenimyxin, and pelgipeptin), the induction of systemic resistance (ISR), hydrolytic enzymes (chitinase, cellulase, and glucanase), and volatile organic compounds. These properties enable *Paenibacillus* strains to suppress the growth of fungi such as *Fusarium oxysporum*, *F. solani*, *Rhizoctonia solani*, *Botrytis cinerea*, or *Colletotrichum gloeosporioides*. Notably, several strains of *Paenibacillus*, including *P. polymyxa*, *P. illinoisensis* KJA-424, *P. lentimorbus* B-30488, and *P. elgii* JCK1400, have demonstrated efficacy in controlling fungal diseases in plants. Importantly, many formulations with *Paenibacillus* strains have already been patented, and some are commercially available, but most of them contain only *P. polymyxa*. Nevertheless, considering the data presented in this review, we believe that other strains from the *Paenibacillus* genus (besides *P. polymyxa*) will also be commercialized and used in plant protection in the future. Importantly, there is still limited information regarding their impact on the native microbiota, particularly from the metataxonomic and metagenomic perspectives. Expanding knowledge in this area could enhance the effectiveness of biocontrol agents containing *Paenibacillus* spp., ensuring safe and sustainable use of biological fungicides.

## Introduction

Plant diseases of various origins cause significant losses in agriculture. Because of their pathogenicity, they reduce both the quality and the size of crop yields [1, 2]. In crops where chemical pesticides are not used, global losses due to plant diseases are estimated to be nearly 20% [2, 3]. Plant diseases can be caused by bacteria, viruses, nematodes, or fungi [4], with fungi being responsible for over 70% of these diseases [5]. The most common phytopathogens include fungi from genera *Alternaria* [6], *Botrytis* [7], *Blumeria* [8], *Magnaporthe* [9], *Verticillium* [10], *Pythium* (oomycetes) [11], *Puccinia* [12], *Rhizoctonia* [13], and *Fusarium* [14]. Until recently, chemical pesticides were the primary means of suppressing fungal diseases [15]. However, increasing environmental and food contamination by fungicides, along with EU directives restricting chemical agents, has driven the development of new eco-friendly plant protection products [16]. Initiatives like the European Green Deal (EGD) and the EU’s 2030 Biodiversity Strategy emphasize the need to incorporate biological agents into agriculture on a larger scale and to expand organic farming [17]. Among eco-friendly solutions, plant growth–promoting bacteria (PGPB) have been used in agriculture for years [18–20]. PGPB can combat phytopathogens through several mechanisms, including the production of antibiotic lipopeptides [21], triggering of induced systemic resistance (ISR) [22], the release of hydrolase enzymes (mainly chitinases) [23], volatile organic compounds (VOCs) [3], and the modulation of the native microbiota in soil or plants that they are inoculated into [16, 24]. One can distinguish various bacterial genera among PGPB exhibiting antifungal traits—*Pseudomonas* [25], *Serratia* [26], and *Streptomyces* [27]. Importantly, due to their spore-forming abilities, *Bacillus* spp. and *Paenibacillus* spp. are particularly intriguing for practical applications.

*Paenibacillus* is a genus of rod-shaped, aerobic, or facultatively anaerobic bacteria belonging to the phylum Bacillota [28]. It had been first identified on the morphology basis as *Bacillus*, but in 1993, after a taxonomic revision, it was classified as a separate genus [29]. Its Latin name, meaning “almost a *Bacillus*,” reflects this history, although the two genera have been now assigned to different families [30].

Members of the genus *Paenibacillus* have been isolated from a vast range of environments, both hot and cold, oxic and anoxic, on land and in water [30]. However, most of them inhabit soil and often associate with plant rhizosphere [31, 32]. Many bacteria belonging to the genus *Paenibacillus* exhibit PGPB abilities, such as nitrogen fixation, phosphate and potassium solubilization, siderophore and phytohormone production, and polysaccharide degradation [28, 30, 31]. Moreover, they play an important role in the suppression of phytopathogens—animals, bacteria, and fungi. However, despite numerous reports and patents (Table 1), the antifungal activity of *Paenibacillus* spp. is still insufficiently described in comparison with *Bacillu*s spp. [33–36], particularly species other than *Paenibacillus polymyxa* that also exhibit antifungal characteristics [37, 38]. A very limited number of biofungicides containing *Paenibacillus* strains are available on the market, and almost no other species than *P. polymyxa* are used in such products (Table 2).

**Table 1:** Examples of patents derived from *Paenibacillus* spp., aiming at bioprotection against fungal phytopathogens

Patent name (number)	*Paenibacillus* strain	Active substance	Fungal pathogen (disease)
Novel strains belonging to the genus *Paenibacillus* and method of controlling plant disease by using these strains or culture thereof (EP 1788074)	*P. polymyxa* BS-0105; *Paenibacillus* sp. BS-0048, BS-0074, BS-0277	Fusaricidin or fusaricidin-like compound	*F. graminearum*, *F. avenaceum*, *F. oxysporum* f. sp. *cucumerinum*, *F. culmorum*, *F. oxysporum* f. sp. *melonis*, *F. oxysporum* f. sp. *lycopersici*, *V. dahliae*, *Phytophthora capsici*, *Ralstonia solanacearum*
Biocontrol agent and pesticide (EP 1079692)	*P. polymyxa* PKB1	2 branched cyclic peptide antibiotics of 8 amino acids	*Leptosphaeria maculans*, *Sclerotinia sclerotiorum*, *Marasmius oreades*, *Pythium pythioides*, *Rhizoctonia solani*, *Fusarium avenaceum*,* Alternaria brassicae*
Peptide antibiotic against *Leptosphaeria*, *Micrococcus*, *Streptomyces*, *Escherichia*; crops, canola (US 6602500)	*P. polymyxa* ATCC 202127 (= PKB1)	Polymyxins (peptide antibiotics)	*Leptosphaeria* spp., *Sclerotinia* spp., *Rhizotonia* spp., *Pythium* spp., *Fusarium* spp., *Alternaria* spp., *Aspergillus* spp., *Sporobolomyces* spp., *Penicillium* spp., *Marasmius* spp.
Bacteria Conferring Bioprotection and/or Biofertilizer Properties (US20240130312)	*Paenibacillus* sp. strain S02	Fusaricidin B	*Fusarium oxysporum* and *Colletotrichum graminicola*
Antifungal *Paenibacillus* strains, fusaricidin-type compounds, and their use (CA2956880)	*Paenibacillus* sp. Lu16774, Lu 17,007 and Lu17015	Fusaricidin A, B, C, D, LI-F06a, LI-F06b, and LI-F08b; fusaricidin-like compounds A1 and B1	*Alternaria* sp., *Botrytis cinerea*, *Phytophthora infestans*, and *Sclerotinia sclerotiorum*
Novel strains belonging to the genus *Paenibacillus* and method of controlling plant disease by using these strains or culture thereof (CA2575769)	*Paenibacillus* sp. BS-0048, BS-0074, BS-0277, *Paenibacillus polymyxa* BS-0105	Fusaricidin A, fusaricidin B	*Colletotrichum* sp. (strawberry anthracnose), *Fusarium* sp.
Mezclas y composiciones que comprenden cepas de *Paenibacillus* o fusaricidinas y pesticidas químicos (ES2959636)	*P. polymyxa* sp. nov. ssp. *plantarum* strains Lu 17,007, Lu 17,015	Fusaricidins; chitinase, cellulose, and amylase	*Alternaria solani*, *Botrytis cinerea*, *Phytophthora infestans*, and *Sclerotinia sclerotiorum*
*Paenibacillus polymyxa* strain ATCC 202127 for biocontrol of bacteria and fungi (US6602500)	*P. polymyxa* ATCC 202127 (= PKB1)	PKB1 antibiotic peptides	*Leptosphaeria maculans*, *Sclerotinia* sp., *Rhizoctonia* sp., *Pythium* sp., *Fusarium* spp., *Alternaria* sp., *Aspergillus* sp., *Sporobolomyces* spp., *Trichoderma* sp., *Penicillium* sp., *Marasmius* sp.
Process for the preparation of an antimicrobial peptide (US20230416312)	*P. peoriae* IBSD35	Antimicrobial peptide peoriaerin IBSD35	Various fungal pathogens
Stabilized fungicidal composition (US20210204550)	*Paenibacillus* sp. strain NRRL B-50972	Fusaricidin A	*Alternaria solani*, *Botrytis cinerea*
*Paenibacillus* strain, antifungal compounds, and methods for their use (US20230234988)	*Paenibacillus* sp. strain NRRL B-50972	Fusaricidin, paeniserine, paeniproxilin (fusaricidin-like compounds)	*Phytophthora infestans*, *Plasmopara* sp., *Uromyces* sp., *Sphaerotheca fuliginea*, *Uncinula necator*, *Alternaria solani*, *Puccinia triticina*, *Botrytis cinerea*
Plant growth promoting rhizobacterial strains and their uses (WO2015114552)	*P. barcinonensis* A10	Possibly: antifungal volatile substances	*Phytophthora capsici*, *R. solani*
	*P. alvei* T-29	Possibly: siderophores, chitinase, antifungal volatile substances	*F. oxysporum*, *F. graminearum*, *R. solani*, *V. dahliae*, *C. coccodes*
Isolated bacteria for the protection of plants against phytopathogenic fungi and bacteria (EP1241247)	*P. polymyxa* VKPM B-7961	Possibly: antibiotics, siderophores, cell-wall lytic enzymes, PGP substances stimulating the immunity response of plants	*Alternaria* sp., *Botrytis cinerea*, *Dactylium dendroides*, *Fusarium graminearum*, *F. oxysporum*, *Helminthosporium sativum*, *Mycogone perniciosa*, *Phomopsis*, *Sclerotinia*

**Table 2:** Examples of agricultural fungicides derived from *Paenibacillus* spp., applied in bioprotection against fungal phytopathogens

*Paenibacillus* strain	Product	Producer
*P. polymyxa* DCF	Fungilitic	ADIUMENTUM (PL)
*P. polymyxa* DCF B/00052	FungiZum	Perma Guard Agro Sp. z o.o. (PL)
Bacteria (including *Paenibacillus* sp.) and fungi	Safe-Grow	Wellcrop Biotech (IND)
*P. polymyxa* KN 03	No typical name	Weifang Heyi Agrochemical Co., Ltd (CHN)
*P. polymyxa*	No typical name	Novobac (Tangsons Biotech) (CHN)
Bacteria (including *P. polymyxa*) and fungi	BACTIM® FERTIMAX	Intermag sp. z o.o. (PL)
*P. azotofixans*	No typical name	Indogulf Bioag (IND)
*P. polymyxa*	No typical name	MarkNature USA)
*P. polymyxa*	No typical name	Henan Haoyuhang Economic&Trade Co., Ltd. (CHN)
*P. polymyxa*	No typical name	Jaipur Bio Fertilizers (IND)

Therefore, the review aims to summarize the current knowledge on the application of *Paenibacillus* spp. in the biocontrol of fungal phytopathogens, identify knowledge gaps, and outline new research directed at improving the antifungal properties of *Paenibacillus* spp.

## Hydrolytic Enzymes

Hydrolytic enzymes play a crucial role in the biological control of fungal phytopathogens by degrading structural components of fungal cells. These enzymes include chitinases, glucanases (hydrolyzing β-1,3- and β-1,6-glucans), and cellulases [39, 40]. Due to chitin being the most abundant and primary component of the cell wall in most fungi, chitinases appear to be the most important enzymes potentially involved in the biocontrol of fungal phytopathogens [41]. Bacterial chitinases are encoded by various genes such as *chiA*, *chiB*, *chiC*, and *chiD* [42, 43], and belong to the family 18 of glycoside hydrolases (GH18) [44]. So far, chitinase production has been detected in many representatives of the genus *Paenibacillus* [41, 45, 46]. For instance, in co-culture analysis, it has been demonstrated that chitinase-producing *P. illinoisensis* KJA-424 (producing three different chitinases) can suppress the growth of *R. solani* [45]. Also, *P. illinoisensis* UKCH21 was also able to suppress the growth of *R. solani* as well as *F. solani* and *S. rolfsii* [47]. In an in vitro assay, also *Paenibacillus* sp. NBR10 was capable of antagonistic reactions to fungal phytopathogens including *R. solani*, *F. oxysporum*, and *Alternaria burnsii* [48]. Besides, a member of *Paenibacillus* spp.—*P. ehimensis* MA2012, able to produce chitinase—reduced the growth of *Colletotrichum gloeosporioides* in vitro [49].

Interestingly, *Paenibacillus* sp. D1 was capable of producing chitinase, which, after purification, exhibited higher stability in the presence of commonly used protectant fungicides (captan, carbendazim, and mancozeb). It suggests that *Paenibacillus* sp. D1 may potentially be applied in combination with reduced amounts of these fungicides [50].

Additionally, several studies have explored the use of *Paenibacillus* spp. strains that produce chitinases in experiments with infected plants. For example, the chitinase-producing strain *P. elgii* HOA73 was able to combat gray mold on tomatoes and, as the authors found out, it exhibited effectiveness comparable to a standard fungicide [51].

Another group of enzymes responsible for degrading components of the cell wall of pathogenic fungi are glucanases. These enzymes belong to the glycoside hydrolase family and are encoded by many genes [52, 53]. Similar to chitinases, glucanases can also be produced by *Paenibacillus* strains. For instance, *P. polymyxa* HX-140 was able to reduce *Fusarium* wilt infection by nearly 56% in cucumber seedlings (greenhouse pot experiment), primarily due to its production of β-1,3-glucanase [54]. Recently, Yang et al. [55] also observed that the β-1,3-glucanase-producing strain *P. polymyxa* PJH16 can be used as an agent against cucumber *Fusarium* wilt (*Fusarium oxysporum f.* sp. *cucumerinum*). Besides, *P. polymyxa* sp. 5L8 (β-1,3-glucanase-producing strain) showed antagonistic activity against phytopathogens such as *Aspergillus aculeatus*, *Bipolaris maydis*, *Bipolaris sorokiniana*, *Cochliobolus heterostrophus*, *F. graminearum*, *Phomopsis chimonanthi*, and *Verticillium dahliae* [56].

Cellulases are a highly diverse group of enzymes classified into many glycoside hydrolase families (e.g., GH5, GH6, GH7, GH8, GH12, GH30, and GH44) and are encoded by over 100 genes [18, 57]. Strains of *Paenibacillus* spp. that produce cellulases can be used to suppress the growth of fungi with cell walls rich in cellulose. For instance, electron microscopic observations revealed that the presence of cellulase-producing *Paenibacillus* sp. B2 causes disorganization of the cell contents of *Phytophthora parasitica* [58]. Moreover, *P. polymyxa* SC09-21, capable of producing cellulases, was able to reduce the symptoms of Phytophthora blight caused by *Phytophthora capsici* in pepper plants (greenhouse experiment) [59]. The strain *P. ehimensis* KWN38, known for its cellulolytic activity among other traits, has been shown to inhibit the growth of *P. capsici* [60]. Also, *Valsa pyri*, the causative agent of Valsa canker, which contains cellulose in its cell wall, can be suppressed by *P. polymyxa* strain Nl4, as evidenced by experiments on pear twigs [61].

Furthermore, several studies highlight the involvement of proteolytic strains of *Paenibacillus* spp. in suppressing fungal growth. Examples include (i) *P. polymyxa* APEC128, which effectively suppressed *Colletotrichum gloeosporioides* and *C. acutatum* (causing apple anthracnose) in harvested apples [62]; (ii) *P. polymyxa* SC09-21, which demonstrated efficacy against *Fusarium oxysporum* f. sp. *radicis-lycopersici* in a tomato greenhouse experiment [63]; and (iii) *P. polymyxa* SeR8 and BR20, which were effective in controlling *Fusarium graminearum* in a wheat greenhouse experiment [64].

## Lipopeptides

Lipopeptides are chemical compounds composed of a peptide chain and a hydrophobic lipid chain [65, 66]. They are key determinants of the antifungal properties of *Paenibacillus* spp. [67]. These compounds exhibit a broad spectrum of antibacterial and antifungal activities through a variety of mechanisms, such as disrupting pathogen cell membranes, inhibiting cell wall biosynthesis, and inducing apoptosis [66, 68]. Their potential as natural plant protection agents, which can reduce reliance on chemical fungicides and promote the sustainability of agroecosystems, is currently intensively studied [16].

**Fig. 1:**
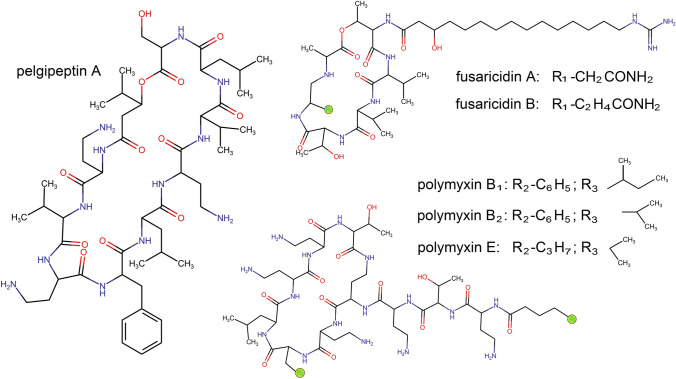
Structural formulae of some non-ribosomally synthesized antimicrobial peptides produced by *Paenibacillus* strains [71–73]

The best-studied antibiotics produced by *Paenibacillus* spp. are primarily non-ribosomally synthesized peptides, including fusaricidin, pelgipeptin, polymyxin B, tridecaptin, and colistin (polymyxin E). There are also ribosomally synthesized peptides such as paenibacillin, paenilan, and paenicidin [69, 70]. The first group of substances is produced independently of RNA, with their presence determined by genes encoding lipopeptide synthetases, among other factors [71]. Some examples of antimicrobial compounds produced by *Paenibacillus* spp., along with their chemical formula, are presented in Fig. 1.

The most common antifungal lipopeptide of the genus *Paenibacillus* is fusaricidin, first detected in *P. polymyxa* KT-8 in 1996 [74]. Fusaricidin is composed of hexapeptide rings containing at least one bond in addition to the amide bonds, with an attached guanidinylated ß-hydroxy fatty acid [75]. Fusaricidin has been detected in *P. polymyxa*, *P. elgii*, *P. tyrfis*, *P. tianmuensis*, and *P. peoriae*, among others [76, 77]. Biosynthesis of fusaricidins is encoded by the fus gene cluster, which consists of eight genes—*fusA*, *fusB*, *fusC*, *fusD*, *fusE*, *fusF*, *fusG*, *fusH*—with the *fusA* gene being crucial as it encodes the protein involved in the synthesis of the main structure of the substance, making it the most important gene in the cluster [78, 79]. To date, more than 20 variants of these compounds have been detected and described, including fusaricidins A, B, C, D, LI-F03, LI-F04, LI-F05, LI-F06, LI-F07, and LI-F08 [78, 80, 81]. For example, fusaricidins A, B, C, and D exhibit considerable activity against fungi (also Gram-positive bacteria) by causing damage to the membrane structure, but show only marginal activity against Gram-negative bacteria [78]. *Paenibacillus* spp. (including *P. polymyxa*) can also produce lipopeptides similar to fusaricidin, such as saltavalin and gatavalin [82]. Additionally, genes encoding iturin A synthetase have been found in *Paenibacillus* sp. strain JDR-2 [83], a surfactin cluster in various strains of *P. polymyxa* [51], and paenilarvin (iturin-like lipopeptides) synthetase genes in *P. polymyxa* HY96-2 and SC2 [84].

The role of lipopeptides in inhibiting the growth of fungal phytopathogens has been documented in a number of studies on *Paenibacillus* spp. So far, fusaricidin has shown activity against fungal phytopathogens belonging to the genera *Fusarium*, *Phytophthora* (oomycetes), *Bipolaris*, *Puccinia*, and *Botrytis*. For instance, *P. polymyxa* E681, studied for controlling *Phytophthora capsici*, was able to produce fusaricidin compounds [85]. After inoculating 3-week-old red pepper plants (*Capsicum annuum* L.) with fusaricidin, the disease severity caused by *P. capsici* was reduced [85]. Moreover, Jiang et al. [77] found noticeable antifungal activity in *P. peoriae* HJ-2 against stem rot (*F. concentricum*) in a *Paris polyphylla* experiment (greenhouse and field conditions); the authors suggested that this activity could be attributed, in part, to the presence of genes encoding fusaricidin synthetase in the genome of *P. peoriae* HJ-2. The study conducted on *P. polymyxa* Y-1 proved that the fusaricidin-reducing *Pestalotiopsis* sp. infection (experiment with *Dendrobium nobile*) primarily inhibits energy production in the respiratory chain and amino acid biosynthesis of *Pestalotiopsis* sp. [86]. Another study on *P. polymyxa* (strain AF01) demonstrated notable activity against *Neoscytalidium dimidiatum* (pitaya canker) and four other pitaya fungal pathogens (*Botrytis cinerea*, *Bipolaris cactivora*, *Fusarium equiseti*, and *Gilbertella persicaria*) [87]. Importantly, after the application of the *P. polymyxa* AF01, a considerable decline in the disease index of pitaya canker was observed in both pot and field experiments on pitaya (*Hylocereus polyrhizus*). The authors revealed that *P. polymyxa* AF01 was capable of producing as many as 13 types of fusaricidins, which directly inhibited mycelial development, spore germination, and germ tube elongation, causing irreversible damage to membrane integrity and cell structure [87].

Furthermore, several forms of fusaricidin were detected in *P. polymyxa* JY1-5. The antifungal activity of the studied strain was demonstrated by an in vivo experiment on tomatoes where a substantial reduction in symptoms of gray mold (*Botrytis cinerea*) was observed [88]. Through mass spectrometry, fusaricidin-type compounds were also observed in *P. polymyxa* TP3; the authors speculate that the compounds are involved in activity against gray mold in strawberry experiments (controlled and field conditions) [89].

Additionally, bacteria of genus *Paenibacillus* may suppress gray mold with other lipopeptides. For instance, *Paenibacillus* sp. B2, capable of producing paenimyxin, inhibited the growth of *Botrytis cinerea* (in vitro) and significantly reduced the abundance of *Xiphinema* index (nematode) in the soil and the formation of gall in grapevine roots [90]. The application of paenimyxin during the inoculation of *Plasmopara viticola* (oomycete-fungus like organisms) leaf tissue also reduced the number of infection sites compared to the application before fungal inoculation. Interestingly, the researchers using the phytosystem of *P. viticola*-grapevine suggest that paenimyxin acts as a biofungicide against oomycete, but does not trigger plant defense [90].

Another group of antibiotics produced by the genus *Paenibacillus* are polymyxins—mostly polymyxins B and E (colistin). Hsu et al. [91] described antifungal activity of polymyxin B against various *Fusarium* species. The main mechanisms responsible for the inhibition of fungal growth are the reduction in conidia germination rates and the damage of the cell membrane [91]. Also, the compound has been observed to reduce *Botrytis* infection in strawberries [92].

Another group of lipopeptides that suppress fungal growth are pelgipeptins [93]. These lipopeptides exhibit similar effects to fusaricidin, primarily disrupting the fungal cell membrane. Kim et al. [93] demonstrated that the pelgipeptin derived from *P. elgii* JCK1400 is effective in controlling tomato gray mold and wheat leaf rust (*Puccinia triticina*).

Members of the genus *Paenibacillus* are also capable of producing polypeptin C, as exemplified by *P. ehimensis* MA2012 [94]. The application of a polypeptin C-producing strain (*P. ehimensis* MA2012) to mature fruits of pepper inoculated with *Colletotrichum gloeosporioide*s resulted in a substantial inhibition of mycelium growth; additionally, the purified compound significantly affected the morphology of the C. *gloeosporioides* mycelium [94].

Finally, it is worth noting that *Paenibacillus* spp., like *Bacillus* spp., may also suppress the development of fungal diseases through the production of iturin or fengicin [95]. Recently, Cai et al. [95] observed a noticeable decline in the disease index of cucumber *Fusarium* wilt (*Fusarium oxysporum* f. sp. *cucumerinum*) after applying iturin- and fengicin-producing *P. polymyxa* hg18.

## Volatile Organic Compounds (VOCs)

VOCs produced by bacteria are also recognized as biocontrol agents [96]. These VOCs are low molecular weight compounds that easily evaporate and disperse at standard temperatures and pressures [97]. The primary chemical groups of VOCs include alcohols, esters, aldehydes, alkenes, alkanes, acids, ketones, benzenoids, pyrazines, terpenes, nitrogen compounds, and sulfur compounds [16, 98]. mVOCs that exhibit antifungal properties include acetoin, benzothiazoles, butylated hydroxytoluene, caryophyllene dodecanal, hexadecanal, N,N-diethyl-1, and 4-phenylenediamine [16, 99–101]. Importantly, as mentioned before, bacterial VOCs can elicit ISR.

Microorganisms that can suppress fungal growth through the production of mVOCs include species from genera such as *Bacillus* [102], *Streptomyces* [103], *Pseudomonas* [104], and *Chryseobacterium* [105]. Also, bacteria of the genus *Paenibacillus* are capable of releasing VOCs [106, 107]. For instance, VOCs released by *P. polymyxa* SY42 showed robust inhibitory effects on three *Fusarium* species—*F. oxysporum* (30%), *F. solani* (30%), and *F. redolens* (22%) [108]. Moreover, in studies conducted on *P. polymyxa* WR-2, 42 VOCs were identified, with seven (benzaldehyde, benzothiazole, dodecanal, hexadecanal, phenol, 2-tridecanone, and undecanal) exhibiting antagonistic activity against *F. oxysporum* [99]. Similar compounds, such as benzothiazoles and butylated hydroxytoluene, were detected in *P. polymyxa* CF05; the strain showed antifungal effects against *Rhizopus stolonifer* (postharvest fruit rot) [101]. Wang et al. [100] also suggested that VOCs secreted by *P. jamilae* HS-26 (N, N-diethyl-1, and 4-phenylenediamine) may be involved in the control of fungi such as *F. oxysporum*, *B. sorokiniana*, and *R. solani*. Other researchers have identified 2,3-butanediol, acetoin, and 2-methyl-1-butanol in *Paenibacillus* sp. UY79 as potential growth inhibitory agents for fungi such as *B. cinerea*, *F. verticillioide*s, *F. graminearum*, *Macrophomina phaseolina*, *Phytophthora sojae*, *Rhizoctonia solani*, and *Sclerotium rolfsii* [109]. Finally, Leon et al. [110] suggested that *Paenibacillus* sp. PNM-50 produces VOCs (presumably 2-firanmethanol, phenylacetonitrile, undecanoic acid, and 2,4-dimethyl pentanol) and could potentially be used to inhibit the growth of *Colletotrichum gloeosporioides*.

## Induced Systemic Resistance

Induced systemic resistance (ISR) is a process by which plants develop broad-spectrum resistance to various pathogens and pests in response to prior stimulation by non-pathogenic microorganisms, such as beneficial bacteria or fungi. It is one of the mechanisms of natural plant immunity, functioning similarly to the animal immune system, although based on different biochemical principles [16, 111]. Bacterial strains exhibiting ISR-inducing features are considered biocontrol agents [16, 112]. Although the precise mechanism of ISR activation by plant growth–promoting bacteria (PGPB) is not yet fully understood, it is suggested that ISR may be triggered by compounds such as lipopeptides, volatile organic compounds, flagellin, lipopolysaccharides, and siderophores [16, 24, 113]. PGPB-derived factors can induce changes in the physiological state of the plant by influencing the metabolite accumulation and the expression of resistance-related genes (e.g., enhance transcription), such as those involved in the biosynthesis of jasmonic acid (JA), including the *lox* (lipoxygenase gene), *aos* (allene oxide synthase gene), *opr* (12-Oxo-phytodienoate reductase gene), and *oxo* (12-oxo-phytodienoate reductase) genes. They also regulate ethyl (ET) levels through genes like *erf1* (ethylene response factor 1).Together, these changes contribute to faster and stronger responses of the plant’s immune system [114].

In terms of *Paenibacillus* spp., Li and Chen [79] used qRT-PCR and suggested that fusaricidin may trigger ISR through SA signal against *Fusarium* wilt of cucumber. Other studies on *Paenibacillus* spp. have also noted that lipopeptides can induce ISR. *Paenibacillus* sp. B2 was capable of boosting plant defense mechanisms through secreting lipopolypeptide elicitor, namely paenimyxin [115]. Real-time PCR assay revealed that, in the endophytic and non-endophytic states, *Paenibacillus* sp. B2 provided ≥ 59% protection against *Septoria* leaf blotch (*Mycosphaerella graminicola*) by triggering ISR via overexpression of *oxo* (oxalate oxidase gene), *pr1* (pathogenesis-related gene), *peroxidase*, *lox*, *aos*, and *gst* genes. Moreover, paenimyxin provided 76% local protection via overexpression of *aos*, *gst* (glutathione S-transferase), *chs* (flavonoid biosynthesis gene), *glu* (glutamate metabolism), *lox*, *pal* (phenylalanine ammonia-lyase—flavonoid biosynthesis gene), and *oxo* genes, and over 80% systemic protection by *chs*. Accordingly, genes involved in the jasmonic acid, flavonoid, salicylic acid, reactive oxygen species, and basal defense pathways appear to play an important role in building immune response against *Septoria* leaf blotch [115]. Earlier, Selim et al. [116], by studying the same strain, demonstrated that *Paenibacillus* sp. B2 strain induces the production of hydrogen peroxide (H_2_O_2_) and increases the expression of several genes related to *Medicago truncatula* defense against *F. acuminatum*. These genes are part of a broader group involved in the biosynthesis of phytoalexins, including those encoding chalcone reductase, phenylalanine ammonia-lyase, chalcone synthase, and genes encoding chitinase (which has antagonistic activity) [116].

Fatouros et al. [117], who conducted a study on the antagonistic effect of *P. alvei* K165 against *R. solani*, *Pythium ultimum*, and *S. sclerotiorum* in lettuce, showed several robust patterns associated with ISR. Following the application of *P. alvei* K165 to plants infected by *P. ultimum*, researchers documented the up-regulation of *pr1.* In lettuce plants infected with *R. solani*, they observed the up-regulation of *lox* and *erf1* (ethylene response factor 1), while in plants infected with *S. sclerotiorum*, the up-regulation of three genes—*pr1*, *lox*, and *erf1—*was recorded. These results suggest that *P. alvei* K165 elicits the salicylic acid (SA)—and the ethylene (ET)/JA-mediated ISR against *Pythium ultimum* and *R. solani*. Additionally, the simultaneous activation of both the SA and ET/JA pathways is proposed for *S. sclerotiorum* [117]. Subsequently, the same researchers conducted a screening of histone acetyltransferase mutants, ChIP assays, and transcriptomic analysis and showed that histone acetylation substantially contributes to the biocontrol activity of *P. alvei* K165 and the establishment of hereditary resistance to *V. dahliae* [113]. The application of *P. polymyxa* K165 induced the expression of immunity-related marker genes and the cinnamyl alcohol dehydrogenase gene *cad3* via the function of histone acetyltransferases. The authors demonstrated that the progeny of plants inoculated with *P. alvei* K165 have enhanced the immunity and increased lignification, which contributes to the hereditary immune response associated with the strain they studied [113]. Similarly, *Paenibacillus* sp. NSY50 was able to considerably up-regulate the expression of the defense system–related genes *pr1* and *pr5* in cucumber roots infected by *F. oxysporum* f. sp. *cucumerinum* at an early growth stage [118]. However, other genes related to the defense response system, including the plant nucleotide-binding site (NBS)–leucine-rich repeat (LRR) gene family, genes coding for phenylalanine ammonia-lyase, and *gst*, were down-regulated following the application of *Paenibacillus* sp. NSY50 compared to plants challenged solely with *F. oxysporum* after 9 days of inoculation [118]. Other studies have also shown that inoculation with bacteria of the genus *Paenibacillus* increases the expression of resistance-related genes, including *pr1*, *pr3*, *pr5*, *pr6*, and *act* (actin) in plants infected with *F. oxysporum* (tomato) [119]. Furthermore, the application of *P. polymyxa* SF05 contributed to the stimulation of the expression of resistance-related genes in leaves and stems, including *opr1* and *opr7* (encoding 12-oxophytodienoate reductases involved in JA biosynthesis) and the previously mentioned *pr1*, in plants infected by *R. solani* [120].

In a related study, Kim et al. [121] observed enhanced defense responses in *Solanum lycopersicum* L. treated with *Paenibacillus terrae* AY-38 by directly measuring salicylic acid (SA) and jasmonic acid (JA).

Interestingly, Dixit et al. [122] reported that the use of *Paenibacillus lentimorbus* B-30488 to suppress *Sclerotium rolfsii* led to increased expression of autophagy-related genes in infected tomato tissues, suggesting an enhancement of disease resistance in tomatoes. Recently, Zhu et al. [123] suggested that a consortium comprising *P. peoriae* SR235 and *Trichoderma yunnanense* SR38 may boost the resistance of *Crocus sativus* to diseases by triggering an immune response. This response is mediated through the increased production of DL-3-phenyllactic acid, 3-hydroxydecanoic acid, and (2S)-2-isopropylmalate—compounds that may bind to the membrane receptors, and inducing local resistance in the plants.

**Fig. 2:**
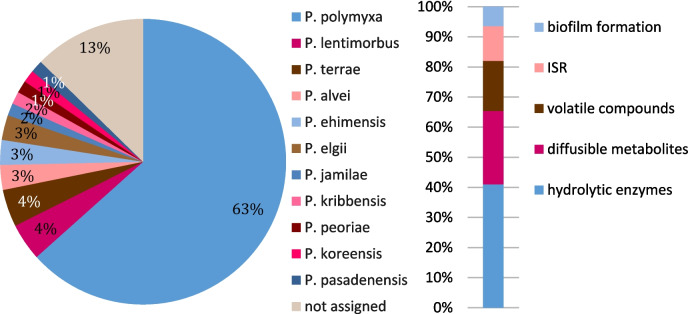
Fungistatic activity of *Paenibacillus* strains; **a** bacterial strains known to suppress fungal growth; **b** main antimicrobial mechanisms and their occurrence

Antimicrobial compounds are produced by a number of *Paenibacillus* species. The vast majority of reports concerns *P. polymyxa*, but nevertheless, at least one-fourth of strains able to suppress fungal growth belong to other species (Fig. 2a). Their activity is related to the release of hydrolytic enzymes, lipopeptides, volatile organic compounds, the stimulation of induced systemic resistance in plants, and—in some cases—to biofilm formation (Fig. 2b). Importantly, a lot of research on *Paenibacillus* strains has been done without determining the trait by which the bacteria suppress fungal growth (Table 3).

**Table 3:** Fungistatic activity of *Paenibacillus* spp. (antifungal factor unknown)

*Paenibacillus* strain	Host plant	Fungal pathogen	References
*P. polymyxa* ShX301	*Gossypium hirsutum* L. cv Ejing-1	*Verticillium dahliae*	[124]
*P. polymyxa* SCE2	–-	*Aspergillus versicolor*	[125]
*P. alvei* K165	*Vitis vinifera* L. cv Soultanina	*Phaeomoniella chlamydospora*	[126]
*P. alvei* K165	*Gossypium hirsutum* L	*Thielaviopsis basicola*	[127]
*P. alvei* K166	*Olea europaea* L. cv Amfissis	*Verticillium dahliae*	[128]
*P. polymyxa* HT16	*Vitis vinifera* L	*Coniella diplodiella* Speq	[129]
*P. ehimensis* KWN38	*Agrostis stolonifera* L. var. *palustris*	*Rhizoctonia solani*	[130]
*P. polymyxa* SCHC33	*Vitis vinifera* L	*Botrytis cinerea*	[131]
*P. kribbensis* T-9	–-	*Botrytis cinerea*, *Colletotricum acutatum*, *Fusarium oxysporum* f. sp. *radicis-lycopersici*, *Magnaporthe oryzae*, *Phytophthora capsici*, *Rhizoctonia solani*, and *Sclerotium cepivorum*	[132]
*P. polymyxa* EBL-06	–-	*Botrytis cinerea*, *Cladosporium cucumerinum*, *Fusarium* spp.	[133]
*P. polymyxa* SG-6	*Citrus reticulata* Blanco cv. Shiyueju	*Penicillium digitatum*	[134]
*Paenibacillus* sp. S19	*Vitis vinifera* L	*Neofusicoccum parvum*	[135]
*P. polymyxa* NMA1017	*Phaseolus* sp. L	*Rhizoctonia solani* RhCh-14, *Pythium ultimum* PyFr-14	[136]
*P. polymyxa* CR1	–-	*Phytophthora sojae* P6497 (oomycete), *Rhizoctonia solani* 1809, *Cylindrocarpon destructans* 2062	[137]
*Paenibacillus sp. P16*	*Brassica oleracea* var.* capitata*	*Xanthomonas campestris* pv. *campestris*	[138]
*P. polymyxa* C1	*Capsicum* sp. L. cv Cabe Besar	*Colletotrichum scovillei SGCR*	[139]
*P. polymyxa* Kp10	–-	*Colletotrichum truncatum* and *C. gloeosporioides*	[140]
*P. polymyxa* GBR-1	–-	*Colletotrichum gloeosporioides*, *Colletotrichum acutatum*, *C. destructans*	[141]

## Potential Side Effects

As demonstrated above, many bacterial strains belonging to the genus *Paenibacillus* can be used as biocontrol agents and have a great potential in sustainable agriculture. They are able to improve soil properties, increase the abundance of beneficial rhizosphere microorganisms, and suppress pathogen growth [142, 143]. However, their antimicrobial activity might also become a threat to soil bacterial and fungal communities. The release of antibiotics and other metabolites may negatively affect the structure and abundance of microbial populations, and indirectly, also plant health [144, 145].

It is also worth noting that some *Paenibacillus* species are known to be harmful to animals, particularly insects. Enthomopathogenic strains are employed to control phytophagous beetles and lepidopterans [30]. At the same time, they pose a threat to other non-target insects, such as honeybees, which are susceptible to bacteria-induced diseases. For example, *P. larvae* is the main cause of American foulbrood [30, 146], while *P. alvei*, *P. dendritiformis*, *P. apiarius*, and possibly other *Paenibacillus* species are associated with secondary infections in European foulbrood [147–149].

In addition, it is important to recognize that *Paenibacillus* strains can act as opportunistic human pathogens [150, 151]. Some microorganisms with great potential in agriculture, such as PGPB species *P. macerans* and *P. massiliensis* [152] or strains exhibiting antifungal activity (*P. alvei*, *P. pasadenensis*), have been reported to cause bacteremia [153]. Therefore, assessing the impact of any *Paenibacillus*-based biocontrol agent on human health and other non-target organisms is crucial before widespread application.

## Conclusions and Future Perspectives

The fungistatic activity of *Paenibacillus* spp. is fairly well documented in the literature, with several patents highlighting their use in the biocontrol of fungal phytopathogens. However, we believe that the mechanisms of action of *Paenibacillus* species are still relatively poorly understood. More comprehensive studies are necessary; as in many cases, the antifungal properties may result from the combined action of several different metabolites produced by *Paenibacillus* spp. A deeper understanding of how these biocontrol traits influence plant health could pave the way for more effective and targeted biological plant protection strategies.

Importantly, there is limited research on the effects of biocontrol strains of the genus *Paenibacillus* on the indigenous microbiota of inoculated soil or plants. To our knowledge, only a few studies have addressed this crucial aspect [144, 145]. Such research is vital because modulating the microbiota—such as increasing the abundance of native taxa responsible for producing biocontrol compounds—can provide additional protection against fungal phytopathogens. Conversely, negatively impacting the diversity of indigenous microorganisms and reducing key taxa in the soil may disrupt ecosystem balance and potentially worsen fungal disease issues.

In conclusion, not only *P. polymyxa*, but also other *Paenibacillus* species hold significant potential for effective fungal biocontrol, and therefore products with such species as *P. peoriae*, *P. alvei*, or *P. lentimorbus* should be introduced to the agricultural market in the near future. However, as emphasized, there is a pressing need for monitoring studies of the native microbiota and other organisms to ensure the safety and efficacy of these biocontrol agents.

## Data Availability

No datasets were generated or analysed during the current study.
